# Ascertaining and classifying cases of congenital anomalies in the ALSPAC birth cohort

**DOI:** 10.12688/wellcomeopenres.16339.2

**Published:** 2021-04-14

**Authors:** Kurt Taylor, Richard Thomas, Mark Mumme, Jean Golding, Andy Boyd, Kate Northstone, Massimo Caputo, Deborah A Lawlor

**Affiliations:** 1 Department of Population Health Science, Bristol Medical School, University of Bristol, Bristol, BS8 2BN, UK; 2 MRC Integrative Epidemiology Unit, University of Bristol, Bristol, BS8 2PS, UK; 3 Avon Longitudinal Study of Parents and Children (ALSPAC), Population Health Sciences, Bristol Medical School, University of Bristol, Bristol, BS8 2BN, UK; 4 Centre for Academic Child Health, Bristol Medical School, University of Bristol, Bristol, BS8 2BN, UK; 5 Department of Translational Science, Bristol Medical School, University of Bristol, Bristol, BS2 8DZ, UK; 6 Bristol NIHR Biomedical Research Center, University of Bristol, Bristol, BS1 2NT, UK

**Keywords:** ALSPAC, birth cohort, congenital anomaly, congenital heart disease, longitudinal cohort, record linkage

## Abstract

Congenital anomalies (CAs) are structural or functional disorders that occur during intrauterine life. Longitudinal cohort studies provide unique opportunities to investigate potential causes and consequences of these disorders. In this data note, we describe how we identified cases of major CAs, with a specific focus on congenital heart diseases (CHDs), in the Avon Longitudinal Study of Parents and Children (ALSPAC). We demonstrate that combining multiple sources of data including data from antenatal, delivery, primary and secondary health records, and parent-reported information can improve case ascertainment. Our approach identified 590 participants with a CA according to the European Surveillance of Congenital Anomalies (EUROCAT) guidelines, 127 of whom had a CHD. We describe the methods that identified these cases and provide statistics on subtypes of anomalies. The data note contains details on the processes required for researchers to access these data.

## Introduction

Congenital anomalies (CAs) occur *in utero* and can be identified prenatally, at birth or during later life. CAs can be defined as structural (e.g. limb reduction defects) or functional (metabolic disorders). The exact cause of most CAs is unknown; however, causes can include single gene defects, chromosomal disorders, multifactorial inheritance, environmental teratogens and micronutrient deficiencies during pregnancy ^1^. Consequences vary depending on the type and severity of the anomaly, but many children and their families experience lifelong complications. Worldwide, at least 3.3 million children under the age of 5 die from CAs each year ^2^. In European countries, including the UK, CAs affect approximately 2–3% of births ^3^. CAs are a major cause of fetal death, infant morbidity and long-term disability. CAs represent a significant public health concern requiring further research around their causes, consequences and long-term implications.

Birth cohorts can be useful for studying the aetiology and longer-term consequences of CAs as they aim to include all births in a defined population over a defined period of time and often follow them into adulthood. Many have the added advantage of recruiting during pregnancy and recording all birth outcomes, whether live or stillborn. This reduces selection bias (in comparison to studies that focus solely on those with CAs or those at risk), provides a comparison group of those without CA from the same underlying population, and with postnatal follow-up allows for all CAs to be identified ^4, 5^. Follow-up supports research into the natural history and impacts of CAs on future health and wellbeing. The latter is important as modern treatments, including advancements in surgery, mean higher proportions of those with CAs now live through to adulthood ^6^. On the other hand, as CAs are relatively rare disorders, statistical power in any single birth cohort is likely to be low, meaning effects will be imprecisely estimated in comparison to case-control studies. Some birth cohorts exclude infants with known CAs from being in the study population or collect information at birth but often then exclude those with known CAs from specific studies ^7^. Other birth cohorts, such as the Born in Bradford (BiB) study, seek data on all CAs, and demonstrate the importance of continuing to identify cases postnatally, for example through linkage to primary and secondary care, in order to identify participants whose clinical diagnoses came later in life ^5^.

In this paper, we describe how we have attempted to identify all cases of major CAs in the UK-based Avon Longitudinal Study of Parents and Children (ALSPAC), a birth cohort which started following participants in the early 1990s. To date, there has been no systematic approach to doing this in ALSPAC. Consequently, it has contributed little to research about CAs ^8^. This is likely because around the time the original women were recruited in pregnancy the routine ultrasound scan screening of all pregnant women was not advanced enough to identify many fetal anomalies ^9^. Additionally, linkage of cohort participants to clinical records was limited as centralised national sources were in their infancy ^10^ and many local datasets remained paper based or in the early stages of digitisation. Here, we demonstrate that combining multiple sources of data including data from antenatal, delivery and neonatal, primary and secondary care health records, as well as parental-reported information can improve case ascertainment. We show that this approach captures more cases than relying on any single data source. We describe how researchers can access these data to undertake research on CAs.

## Methods

### Aims

To: (1) combine a range of data sources to ascertain cases of major CAs in the ALSPAC birth cohort, with a specific focus on congenital heart diseases (CHDs) and (2) code cases of CAs with International Classification of Diseases (ICD) codes (version 10) according to the European Surveillance of Congenital Anomalies (EUROCAT) guidelines ^11^. 

The focus on CHDs reflects the specific research interests of KT, MC and DAL and because CHDs are the commonest form of CAs. However, the available data provide coded data on all CAs and descriptions of their origin (in case researchers want to exclude cases that were only identified in one source or those from a specific source).

### Cohort

ALSPAC is a prospective birth cohort, which was devised to investigate the environmental and genetic factors of health and development. Detailed information about the methods and procedures of ALSPAC is available elsewhere ^12– 14^. In brief, pregnant women with an expected delivery date between April 1991 and December 1992, residing in and around the city of Bristol, UK were eligible to take part. The initial number of pregnancies enrolled is 14,541 (for these at least one questionnaire has been returned or a “Children in Focus” clinic had been attended by 19/07/99). Of these initial pregnancies, there was a total of 14,676 fetuses, resulting in 14,062 live births and 13,988 children who were alive at 1 year of age. When the oldest children were approximately 7 years of age, an attempt was made to bolster the initial sample with eligible participants who had failed to join the study originally (i.e. any child born during the same years and in the same geographical area that defined the original cohort). As a result, for all ALSPAC variables collected from the age of seven onwards there are data available for more than the 14,541 pregnancies mentioned above. The total sample size for analyses using any data collected after the age of seven is 15,454 pregnancies, resulting in 15,589 fetuses. Of these 14,901 were alive at 1 year of age ^14^.

In 2012 recruitment of the next generation (children of the original children born in the early 1990s began) and since then we have described the generations as ALSPAC-G0 (women recruited during pregnancy in the early 1990s and their partners), ALSPAC-G1 (the index children of those women who have been followed since birth) and ALSPAC-G2 (the children of ALSPAC-G1 and grandchildren of ALSPAC-G0) ^15^. This data note is about ascertaining and coding CAs in the ALSPAC-G1 cohort. Data on CAs in G2 are being, and will continue to be, prospectively collected, but currently there will be very few cases amongst the ~1000 G2 participants that have been recruited. All three generations have continued to be followed via questionnaires, research clinics and record linkage. The study website contains details of all the data that is available through a fully searchable data dictionary ( http://www.bristol.ac.uk/alspac/researchers/access/). Ethical approval for the study was obtained from the ALSPAC Ethics and Law Committee and the Local Research Ethics Committees ( http://www.bristol.ac.uk/alspac/researchers/research-ethics/). Consent for the use of data collected via questionnaires and clinics was obtained from participants following the recommendations of the ALSPAC Ethics and Law Committee at the time. All G0 and G1 participants have been informed about the study’s intention to link to and use their routine health records in the study’s research program. Participants are free to object to this use of their records, and the records of those objecting have not been used in this research. When it becomes practicable, explicit consent for linkage to health records is collected (e.g. at study assessment visits). The use of National Health Service (NHS) records in this way has approval from a Health Research Authority (HRA) Research Ethics Committee and the HRA Confidentiality Advisory Group.

### Data sources and methods of obtaining CAs from them

Five data sources were used to identify children with CAs in ALSPAC ( Table 1). Four of these were able to identify any CA, one (data source 2) was specific to CHDs. We included diagnoses made at any age. Restricting diagnoses to a specific age bracket could lead to incomplete case ascertainment ^5^.

**Table 1.** Data sources used to identify cases of congenital anomalies in ALSPAC.

#	Data source	Data collection method	Description and data coverage
1	NHS Primary Care records	Record linkage to Primary Care	Linkage of ALSPAC participants to primary care records. Last extract was October 2016. Capability to capture any CA diagnosed on an ALSPAC participant registered with a participating GP in England/Wales between 1990 and 2016. Further extracts will continue to be made.
2	Paediatric cardiology & cardiothoracic surgery records	Record linkage to paediatric cardiology & cardiothoracic surgery records	The *HeartSuite* patient management system is designed specifically for paediatric cardiology and cardiothoracic surgery. It covers data on diagnoses and procedures between 1992 to 1994 and 2002 to 2019 for a regional referral centre. It would include ALSPAC participants’ residing in and around Bristol who had cardiac/ cardiothoracic surgery or procedures such as catheterisation at the UHBT during the periods covered. Data were provided by UHBT, in November 2019.
3	Data on fetal, infant and child deaths	Birth notification system, ONS, post- mortem reports.	Includes data on fetal deaths of gestation 20 weeks or more in England, Scotland or Wales, including spontaneous and therapeutic abortions for malformations or genetic defects, and deaths of livebirths up to ~104 weeks of age. Data were captured from multiple sources including: The birth notification system of deaths to livebirths in Avon, the Office for National Statistics (ONS), post-mortem reports and the regular clinical discussions of all such deaths in the two major maternity hospitals. This provides the ability to capture CAs that resulted in antenatal or early postnatal death, which might not be captured in other sources.
4	Diagnoses from Avon Child Health Services	Diagnoses from Avon Child Health Services	CAs from Child Health (formerly known as Avon Child Health Services). These data cover the Avon region from December 1990 to February 1993 and would identify children diagnosed at any postnatal age during that period.
5	ALSPAC	i. Antenatal, labour and neonatal records ii. Questionnaire completed by research nurses iii. Participant’s mother self-report	i. *Abstractions from clinical records –* This ** database comprises detailed abstractions from the clinical records covering midwife, obstetrician, paediatric and additional (e.g. blood test results and ultrasound scans) entries from the antenatal, intrapartum and first two weeks of the postnatal period. Abstractions were conducted by ALSPAC employed research nurses on different subgroups. These included several clinical subgroups (e.g. preterm births and multiple pregnancies) as well as a random sample. In total, detailed data has been extracted from 8,369 ALSPAC-G0 pregnancies. In addition, extracted text data with descriptions of all abnormalities of the fetus and neonate were available for 6,343 ALSPAC-G1 fetuses and infants with known birth outcomes and used in this data note. ii. *Neonatal admissions questionnaire -* For each neonate (<28 days of age) admitted to hospital, a detailed questionnaire was completed by a neonatal nurse. In total, 994 questionnaires were completed. Of these, all but 5 were from the two main hospitals in Bristol at the time. iii. *Child-based questionnaires –* KT undertook a search of the text answers from ALSPAC parent (mostly mothers) completed child-focused questionnaires between birth and ~14years. These data would only include G1 participants whose mothers (or another main carer) filled in and returned at least one child-based questionnaire. Questionnaires were searched for key words relating to CAs in response to general questions about the child experiencing diseases, being admitted to hospital, outpatient investigations or a free text space at the end of each questionnaire that carers were invited to use for any other information they thought would be valuable to the study.

Abbreviations: ALSPAC, Avon Longitudinal Study of Parents and Children; CA, congenital anomaly; GP, general practice; NHS, National Health Service; UHBT, University Hospital’s Bristol Trust; ONS, office for national statistics.

***NHS Primary Care Records.*** ALSPAC have established a linkage between participants and their information on the NHS Patient Demographic System (PDS): the national patient register for England, Wales and the Isle of Man. This linkage provides a participant’s NHS ID number and can also be used to identify which General Practice (GP [primary care]) a participant is registered with. The NHS provides free primary and secondary health care to all UK residents. Access to secondary care is via referral from primary care and even when someone has care from a private provider a discharge note will be sent to their general practitioner. Therefore, NHS record linkage will provide health data for the vast majority of the population. It is possible that some participants were or are not registered with a GP, although, we would expect this to be a small minority. To date, ALSPAC have extracted primary care information in two batches:

(1) In 2013 a pilot exercise was conducted, which aimed to extract the records of 2,806 G1 participants registered in 523 primary care practices across England and Wales. ALSPAC gained approval from 290 of these practices to extract life-course GP coded records. These were extracted by EMIS Health Ltd or Apollo Ltd clinical software system providers. This resulted in the extract of 2,249 participants records from 180 practices (the high level of achieved participant coverage reflects that the 180 practices disproportionately included those with high numbers of ALSPAC participants, including those in and around the city of Bristol) ^16^.
(2) In 2016 an additional extract was conducted to extract the records of 11,955 G1 participants from participating practices in the Bristol, North Somerset and South Gloucestershire (BNSSG) clinical commissioning group (CCG), which has the same geographical coverage as the ALSPAC catchment area. This resulted in the extract of 11,087 participants records ^17^. This second extract included most, but not all of the participants in the 2013 pilot, meaning that the final number of ALSPAC-G1 participants (i.e. the participants considered in this paper) with primary care data is 11,810.

For the data described in this manuscript, the majority of primary care records which contributed to our case definition were those extracted from BNSSG GPs in 2016, when participants were aged ~26 years old. There were a small number of additional extracted records from across England and Wales taken in 2013 when participants were aged ~23. However, not all participants will have complete records up to the date of the extract (record loss can have occurred during any of the following: (i) transferring paper-based to electronic records; (ii) when participants move practice; (iii) if practices change record keeping software systems; or (iv) during any amendments made to electronic records made by health professionals). It is also important to note that ALSPAC do not have the governance approvals to extract linked health records for participants who died before the age of 18. In total, there were data on 11,810 participants linked with at least one record, with approximately 3.5 million coded entries in total. We compiled a list of GP Read Codes (the health coding system used in primary care in the UK) used to code diagnoses (see *Extended data*, Table S1) ^18^ to narrow down the dataset with the aim of identifying cases of CAs. In total, this minimized dataset included 1,513 participants, with 4,626 coded entries.

***Paediatric cardiology and cardiothoracic surgery records (HeartSuite).*** HeartSuite is a fully integrated patient management system designed specifically for paediatric cardiology and cardiothoracic surgery. It includes records of paediatric cardiology and cardiothoracic surgery undertaken at University Hospital’s Bristol Trust (UHBT, previously only known as Bristol Royal Infirmary). The data was sought from the UHBT cardiac team through the UHBristol Congenital Cardiac Services Information Analyst and Clinical Data Team. NHS numbers were provided to ensure accurate capture of records after the widespread adoption of the modern NHS number in 1996 (i.e. 5 years after the birth of the oldest ALSPAC-G1 participant). However, some of the medical records pre-dated the advent of NHS numbers and so we used other proabable identifiers to link to these. The probable identifiers used were: ALSPAC-G0 (parents) and -G1 names, dates of birth and addresses (at recruitment and subsequently when participants moved). Many individuals had multiple records in order to capture changes in address or even name. The identifiers included not only the child’s details but also, where possible, the mother’s details because the antenatal, perinatal and very early post-natal tests were performed before the child was given a name. A total of 48,326 records were provided for 12,338 individuals. Early electronic records from UHBT, the STORK maternal and delivery database, contained the individual hospital numbers for each mother and child from 1991–92. These were provided back to UHBT, however, the record system had changed at some stage between then and now and so these were unfortunately not of any benefit. It was unclear which address was held by the HeartSuite database and so this necessitated that all known addresses of each member of the ALSPAC cohort be provided so as to maximise the possibility of generating a match between the databases, although the risk of duplication needed to be accounted for. All transfers of data were performed using AES-256 (a 256 bit) encryption and password protected through a secure data portal.

 The data was provided by UHBT in November 2019 and included all matches found up to that date. There were 377 events, relating to 303 individuals, the majority of which (93%) were a full match including NHS number and the remaining 7% were matched using the probable identifiers mentioned above (the IDs for the records matches using probable identifiers can be made available to researchers using the data if required). There were 11 events between 1 ^st^ April 1992 and 31 ^st^ March 1994 with the remaining 366 events identified after January 2002 (though it should be noted that no pediatric surgery was undertaken in Bristol between these two time periods). UHBT started using Heart Suite in May 2009 and the Bristol Royal Hospital for Children in December 2004 (some previous diagnoses and procedure data from a previous system called Cardiobase were obtained which went back a further ~6 years). Of these matched records, 68 had details in the diagnosis section (including conditions such as CHDs, benign murmur, chest pain and family history of heart condition). The remainder had no diagnosis provided and may have been tested for a suspected cardiac issue, but no problem found. Participants who had CHD but who did not have surgery/a procedure or those treated at a different hospital would not be included. UHBT is a regional referral centre for paediatric vascular surgery with no other hospital in the South West region providing this over the period covered by HeartSuite. It is possible some CHDs may have been detected at a very young age but were unable to be successfully treated and therefore not survivable, this may have excluded some of the early and more severe CHDs from being matched via the HeartSuite database. However, it is plausible that these cases would be identified by the fetal and child deaths data source described below.

***Fetal and child deaths.*** We wanted the data on CAs in ALSPAC-G1 to be as comprehensive as possible, and as CAs are a cause of fetal and early child deaths we obtained data on miscarriages, terminations, fetal deaths and deaths in the first years of life. Presence of malformations, chromosome abnormalities or genetic defects were recorded whether or not they were thought to be the cause of death or reason for termination. These data came from multiple sources: (i) ALSPAC were notified by the Birth Notification System of deaths (including still births) within Avon. Whenever a baby had died outside of the Avon Health authority area, this system was notified, therefore meaning ALSPAC would have obtained information about any baby who had died in the first year of life. (ii) All deaths occurring in England and Wales were notified to the study by the Office for National Statistics (ONS). Death certificates were provided with these notifications. ALSPAC also had an arrangement to obtain any deaths that might have occurred in Scotland. (iii) Chromosome abnormalities in some of the fetal or early childhood deaths, as well as the survivors were identified via the Cytogenetics laboratory at Southmead Hospital, who analysed samples in the South West region where a chromosomal abnormality was suspected or in those with a family history. The way that these data were collected were by ALSPAC team members visiting the cytogenetics department periodically. The records were all classified according to date of birth and details were recorded. Linkage was then independently performed for any that may have been enrolled in ALSPAC.

Professor Jean Golding, who established the ALSPSAC study, was responsible for obtaining details from the clinical records, post-mortems and death certificates and summarising these in a single document. The deaths were classified according to the system involved (nervous system, chromosomal, renal, CHD, syndrome, other, genetic). Information used for the classifications has relied on post-mortem and clinical evidence. Classes of perinatal death were based on a scale adapted from the Wigglesworth classification ^19^. The Wigglesworth classification is one that, in addition to major malformations, classifies the deaths according to when the death mainly occurred or was initiated (i.e. antenatal; intrapartum (including livebirths dying of asphyxia) and features associated with preterm delivery to a livebirth. There was a miscellaneous group into which deaths that did not fall into these categories was put. If a baby born at 29 weeks died after 6 months having been suffering from immature development throughout, he/she would still be classified as a death due to preterm delivery.

***Diagnoses from Avon Child Health Services.*** Data from the congenital malformation records of the NHS Avon Child Health Services (‘child health’), who provided early years community health care services, such as school-based vaccination programmes in the ALSPAC catchment area, were linked to existing ALSAPC-G1 participants data. Only the records of children with one or more recorded CA were linked. This data source includes cases diagnosed between December 1990 and February 1993 (the date of birth range for the eligible study sample) in those living in the original ALSPAC catchment area. Diagnoses were originally reported as categories depending on the bodily system affected as well as diagnoses as free text and were given ICD codes as part of the derivation of a comprehensive set of CA data for this report (see application of ICD codes below). The linked file contained 129 children.

***Sources derived from the ALSPAC cohort***

*(i) Antenatal, labour and postnatal (first 2 weeks) records*

The database comprises detailed extractions from the clinical records covering midwife, obstetrician, paediatric (almost every baby was examined by a paediatrician) and additional (e.g. blood test results and ultrasound scans) entries from during the antenatal, labour and first two weeks of the postnatal period ^20^. The data source used in the present data note comprised 6,343 babies or fetuses from the overall original ALSPAC cohort with a known birth outcome. The source was derived from all the free text in section F: ‘The Liveborn Baby – at Delivery’, from the ‘Delivery Questionnaire’ which is available to view on the ALSPAC website (ALSPAC_DataDictionary.zip\ALSPACDataDictionary\quest_pdf\other\delivery.pdf). Free text descriptions of CAs were initially abstracted by a clinical geneticist according to ICD classification.

*(ii) Neonatal admissions questionnaire*

For each neonate (<28 days of age) admitted to hospital, whether to a Special Care Baby Unit, the Children’s Hospital or elsewhere, a detailed questionnaire was completed by a single neonatal research nurse working for ALSPAC. The questionnaire was first developed by the neonatal paediatrician Dr Heather White for use in Special Care Baby Units by the Jamaican Perinatal Morbidity and Mortality Survey of Jamaica ^21, 22^. In total, there were 994 completed questionnaires. Of these, 989 were from the two main hospitals in Bristol at the time (Bristol Maternity Hospital and Southmead). The locations for the remaining five were ‘elsewhere’ with the exact location not reported on the questionnaires that were examined. In total, 60% of admissions were male and 95% were alive at discharge. We searched through each questionnaire separately and retrieved all cases of reported CAs and assigned ICD-10 codes.

*(iii) Child-based questionnaires*

KT systematically searched questionnaire data completed by the main caregiver of the ALSPAC-G1 (for most participants the mother) in relation to the children covering the period April 1991 to December 2006 (corresponding to ALSPAC-G1 ages 1 month to 166 months). This consisted of searching free text responses from questions, mostly in relation to the health of the child. All of the questions used are listed in *Extended data*, Table S2 ^18^ and can be linked back to the ALSPAC questionnaires which are available on the website ( http://www.bristol.ac.uk/alspac/researchers/our-data/questionnaires/). In total, we used questions from 21 questionnaires. Response rates varied for each questionnaire, ranging from 88% completion for the first one sent at 1 month to 47% completion for the final child-based questionnaire that we considered sent at 166 months. Response rates for all 21 questionnaires can be found in the *Extended data*, Table S2 ^18^. We developed a search strategy of key terms for CAs and corresponding author (KT) applied this to the text fields. He then read a small subsample of these fields to see what proportion of cases might be missed by this search (e.g. because of incorrect spelling) and update the search with the additional (misspelt) terms. This process was repeated until it was felt all cases had been identified. The search strategy can be found in the *Extended data*, Table S3 ^18^.

### Application of ICD-10 codes to identified CA cases

In this section we describe the methods used to assign ICD-10 codes to data from the 5 data sources described above. CAs were grouped by system affected. A child could contribute to more than one system group when they had been diagnosed with multiple CAs. The ICD-10 codes used to define cases can be found in the *Extended data*, Table S4 ^18^.

The EMIS and Apollo primary care data assigns any diagnosis a clinical term version-2 (CTV2) medical ‘Read Code’ as well as a SNOMED clinical term (CT) code. We mapped SNOMED CT codes to ICD-10 codes using the NHS digital SNOMED CT browser (SNOMED International 2017 v1.36.4, https://termbrowser.nhs.uk/). The cross-mapping of SNOMED to ICD-10 is vulnerable to discrepancies due to multiple codes sometimes presenting as a possible match. To account for this, we used best judgement with the data we had by matching the text diagnosis to the ICD-10 code text as closely as possible. There were no instances where we could not find a probable match. HeartSuite data was partially provided with ICD-10 codes. In some instances, there was a text diagnosis without an ICD-10 code. In these cases, KT assigned an ICD-10 code based on the text diagnosis. The data on fetal, infant and child deaths were provided with detailed text on the anomaly present in each death. From this text, KT assigned ICD-10 codes to CA cases. Diagnoses from child health were originally categorized by subgroup with text of the specific diagnoses. KT assigned ICD-10 codes based on the subcategories and text. The ALSPAC delivery questionnaire data was initially assessed by a clinical geneticist who assigned ICD-10 codes based on free text descriptions. For neonatal and child-based (self-report) questionnaires, assigning codes was initially done by KT. In the first instance he grouped text diagnoses by organ or system (e.g. congenital heart disease). Any uncertainty was checked with MC and DAL. Sub-types were then assigned where possible by KT in discussion with MC and DAL.

**Figure 1.**
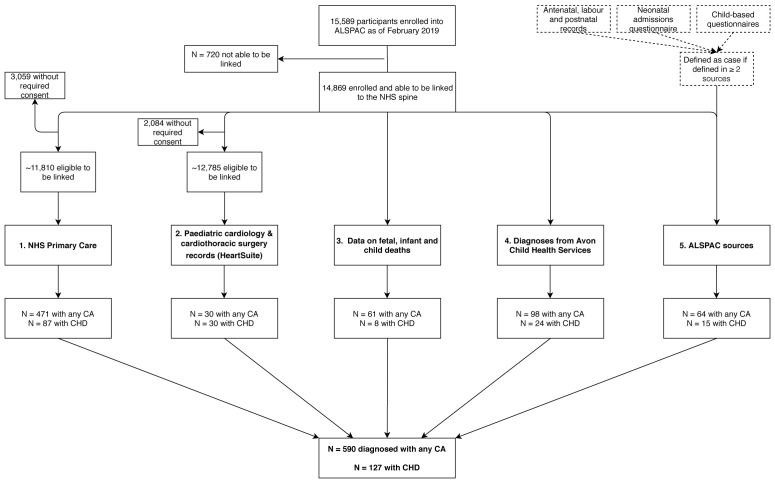
Flow diagram illustrating the multiple sources used to formulate the cases of major congenital anomalies in the ALSPAC cohort. All 30 CA cases within HeartSuite had a CHD diagnosis. Note that the potential capture population for each source may differ and cannot be definitively quantified. Abbreviations: ALSPAC, Avon Longitudinal Study of Parents and Children; CA, congenital anomaly; CHD, congenital heart disease; NHS, National Health Service.

 Once we had ICD codes assigned to all three ALSPAC data sources, we then explored the overlap. Some of the reports in the ALSPAC questionnaires may be less reliable than those from other sources, such as the primary care linked data. For example, either the caregiver or we may have misattributed an abdominal problem that is not a CA to CA status. Therefore we a priori decided that we would only include cases where the same case (at an organ or system level) appeared in at least two of the questionnaires ( Figure 1). Of all of the participants with at least one ICD-10 system/organ code at the end of the initial assignment (N = 672), 64 (9.5%) appeared in at least two of the questionnaires. These (including which questionnaires they were identified in and the remaining 608 (90.5%) that only appeared in one questionnaire are shown in the *Extended data*, Tables S5, 6 ^18^, including which organ/system they came under. To test the assumption that those only found in one questionnaire were more likely to be false positives, we checked how many were defined as a case in the primary care dataset. Of those that appeared in one questionnaire, 21% were a CA case in the primary care data. Of those that appeared in two questionnaires, 50% were a CA case in the primary care data. We labelled the 608 that appeared in one questionnaire as ‘possible CAs’. This variable will be made available to researchers that use the data described in this data note. We have not included these 608 participants with possible CAs in the following sections presenting results (overlap and description of population).

### Overlap across cases and case definition in ALSPAC

We considered an ALSPAC-G1 participant to have a CA if they were identified in any of the 5 sources for our total of ‘any CA’ ( Figure 1). For specific types of CA, these were also defined as occurring in a participant if there was evidence from any of the 5 sources. This is a liberal approach that we hope will minimize false negatives (i.e. missed cases). It might mean that we have included some false positives. We demonstrate overlap between the sources (using a Venn diagram) and future uses of the data will be able to select which sources they use (see *Data access statement*).

## Description of population

**Figure 2.**
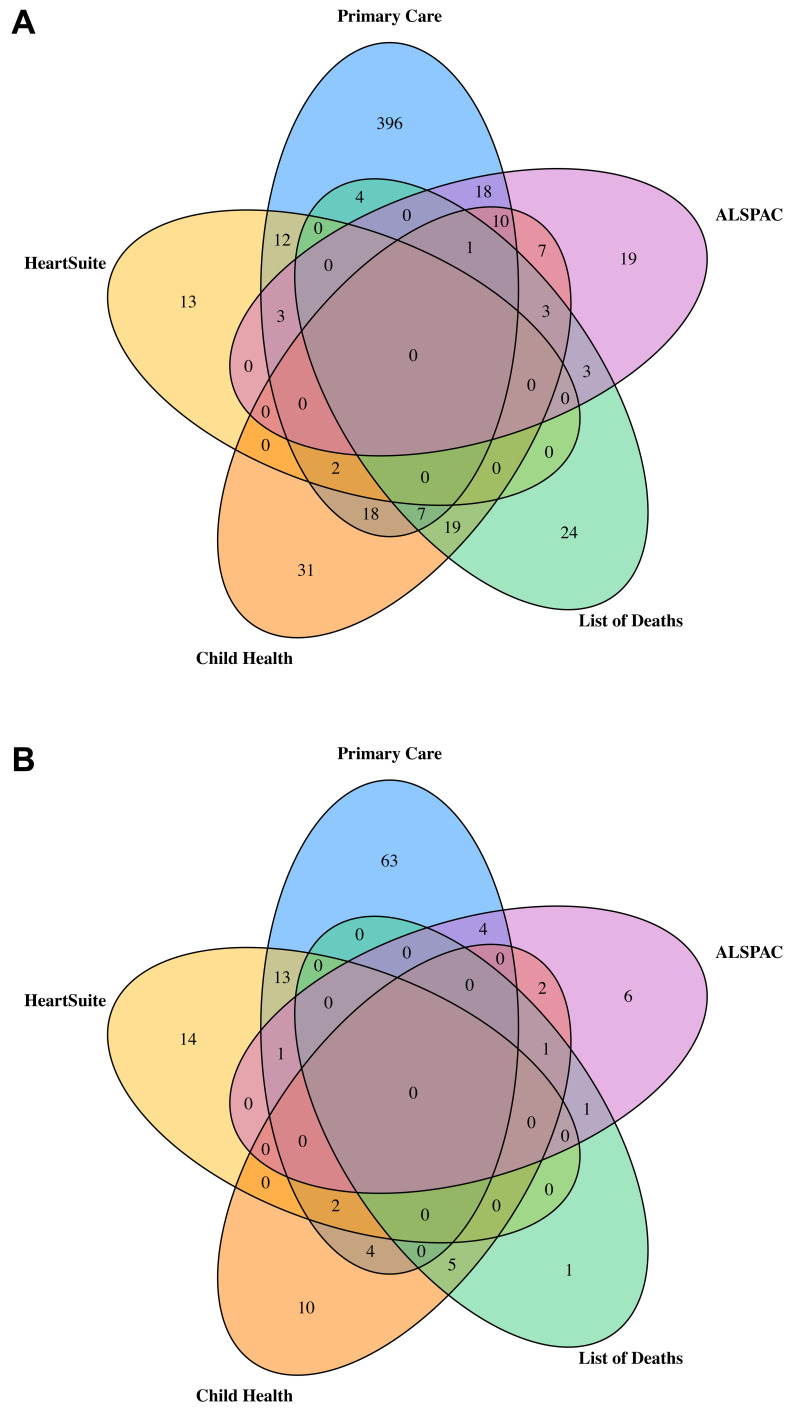
Venn diagrams illustrating the overlap between the 5 data sources for any major congenital anomaly ( **A**; total n = 590) and any congenital heart disease ( **B**; total n = 127) as defined by EUROCAT.

In total, 590 ALSPAC participants were identified as having a CA with a prevalence of 385.5 per 10,000 live births (calculated using 14,791 as the total number of live births for ALSPAC). Of these 590 participants, 151 (25.6%) had a CA occurring in the presence of other anomalies. Figure 2A is a Venn diagram of the number of CA cases from each data source and how they overlap. Primary care data provided the largest number of cases with 471 of the 590 being identified via linkage to primary care. Of the 471 identified via primary care 82 were also identified in at least one other data source. The HeartSuite database contained 30 cases of any CA, all of which had CHD, whilst the mortality data included 61 cases. The child health services data source identified 98 cases and the ALSPAC data source (after limiting to the cases found in at least two of the sub-data sources) included 64 cases. Figure 2B provides the numbers for CHDs only. Of the 127 CHD cases, 87 were identified in the primary care data, with 24 of these also being identified in at least one other data source. Of the 30 cases of CHD identified by HeartSuite, 16 cases were also identified in at least one other data source. The list of deaths contained 8 cases of CHD, child health included 24 cases of CHD and the ALSPAC data source contained 15 cases of CHD. For those 8, 24 and 15, the number of cases found in at least one other data source were 7, 14 and 9, respectively.

Table 2 reports the total number of anomalies in each subcategory and compares the prevalence in ALSPAC to the EUROCAT recorded prevalence for CAs from full European registries between the years 1990–1992 ^23^ (see reference and *Extended data*) ^18^.

**Table 2.** Total numbers of congenital anomalies, numbers in those live born and prevalence per 10,000 live born in ALSPAC-G1 participants (total N live born = 14,791 of the 14,869 enrolled and linkable [see Figure 1]).

Anomaly subtype ^a^	Total N (N born alive) ^b^	Prevalence per 10,000 live births	EUROCAT prevalence per 10,000 live births ^23^
Any CA	590 (570)	385.3	205.7
CHD	127 (119)	80.5	56.0
Nervous system	18 (15)	10.1	13.5
Respiratory	5 (5)	3.4	1.9
Orofacial clefts	16 (16)	10.8	14.2
Eye	29 (29)	19.6	5.6
Ear, face, neck	^*^	^*^	5.7
Digestive system	16 (14)	9.5	20.3
ABWD	^*^	^*^	2.7
Urinary	48 (44)	29.7	28.6
Genital	64 (64)	43.3	10.9
Limb	197 (196)	132.5	48.4
Other	60 (57)	38.5	-
Chromosomal	42 (39)	26.4	15.8
Teratogenic/genetic syndromes, microdeletions and chromosomal abnormalities	67 (63)	42.6	-

Abbreviations: CA, congenital anomaly; CHD, congenital heart disease; ABWD, abdominal wall defects; ABWD, abdominal wall defects; * used when there were fewer than 5 cases in a given category all of these would have prevalence per 10,000 <3.4. a ICD codes used to define subtypes can be found in the Extended data ^18^. b We have included all cases in ALSPAC including whether they resulted in a fetal death. We give the number live born in brackets and this is used to estimate live born prevalence for comparison with EUROCAT results. Minor anomalies according to EUROCAT are not included. Numbers represent cases of congenital anomalies; if a child had multiple anomalies affecting different systems, they would contribute to more than one category. Each child could contribute to each category once.

**Figure 3.**
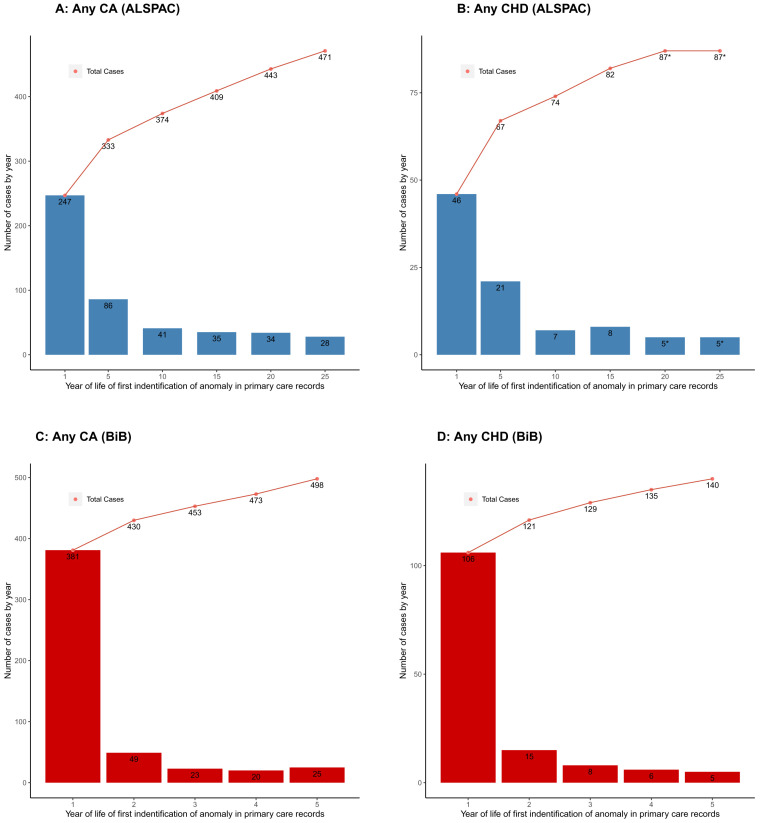
Showing the number of congenital anomaly (CA) ( **A**) and congenital heart disease (CHD) ( **B**) cases at different ages using linked primary care data in ALSPAC. Age cut-offs are diagnoses in first year of life and then up until age 5, 15, 20 and 25. The age-25 column includes all diagnoses from the 2016 primary care extraction; therefore, some participants may be slightly older than 25, but younger than 26. Numbers are presented at the child level, so if a child had multiple anomaly diagnoses, they would only be counted once (at the time of their first diagnosis). For comparison, ( **C**) and ( **D**) show corresponding estimates for any CA and any CHD respectively from the Born in Bradford cohort primary care extraction up until age 5 (Adapted with permission from Bishop *et al.* (2014) ^5^. Bars show the number of cases in each age category and points show the cumulative number of cases. *Cell values <5 are suppressed for disclosure control purposes (may include 0).

It is possible that EUROCAT underestimates the total prevalence of CAs because the age range for data capture is capped at or before age 1 for 61% of the full registries and only for 28% does it go to age 5 years or beyond. By comparison, the inclusion of primary care linkage in our sample means we have included cases that are diagnosed in participants up to their early-/mid-20s and it is notable that primary care linkage provides the highest proportion of ALSPAC cases. Using just the primary care linked data in ALSPAC shows an increase in new cases of CAs after age 1, with the rate of increase with age slowing but still continuing up to early 20s ( Figure 3A), with a similar illustration for CHDs ( Figure 3B). Previous analyses in the BiB cohort also demonstrated a marked increase in numbers of CA cases diagnosed after 1 year of age through record linkage to primary care data up to when participants were aged 5 years ^5^ ( Figures 3C, D). It is possible that the liberal definition that we have used here, defining a case as being from any of the five data sources, may mean we have overestimated the prevalence in ALSPAC. However, as can be seen from the description of the different data sources above and summarised in Table 1, the different data sources cover different geographical regions at diagnosis, time periods and have different sources of missing data. If we were to exclude a particular data source, we would have missed some true cases. It is also possible that other factors that influence the risk of CAs differ between pregnancies in the early 1990s in the South West of England and EUROCAT data for pregnancies for the same time period across the whole of Europe.

CHDs are the commonest form of CA and in Table 3, we report numbers for CHD subtypes. As expected, septal defects make up a large proportion of cases with 82 (65%) CHD cases having a septal defect, slightly higher than recent global estimates of around 55% ^24^. Of the 127 CHD cases, 35 (28%) were classed as severe, which is higher than found in the Norwegian National birth cohort, which recruited pregnancies between 1999 and 2008 and found that 19% of CHD cases were defined as severe using a similar classification system ^25^. The prevalence of CHDs in ALSPAC is similar to other European birth cohorts. In recent work involving 7 European birth cohorts, we have shown that the prevalence of CHD was close to 1% in most cohorts, with the lowest with 0.4% and the highest with 1.4% ^26^. Differences in case ascertainment could be one of a number of possible explanations for the slight differences in prevalence estimates.

**Table 3.** Congenital heart disease subtypes.

CHD subtypes	N
Severe CHD ^a^	35
Non-severe CHD	92
Any septal defect	82
Atrial septal defect	20
Conotruncal ^b^	6
Isolated CHD ^c^	110
CHD with other CAs ^d^	17
CHD associated with syndrome ^e^	13
Any CHD	127

Abbreviations: CHD, congenital heart disease; CA, congenital anomaly. ^a^ According to EUROCAT. See supplementary table X for ICD codes. ^b^ Tetralogy of Fallot, transposition of great arteries, truncus arteriosus, double outlet right ventricle. ^c^ Those diagnosed with a CHD (or multiple CHDs) and no other congenital anomalies ^d^ CHDs cooccurring with other congenital anomalies. ^e^ CHDs diagnosed with other syndromes (see ‘probable cause’) in Table 2 above.

## Strengths and limitations of the data

A key strength of this dataset is the combination of multiple sources of data to identify cases. This enabled the capture of additional cases that might have otherwise been missed. That said, our results indicate a strong reliance on record linkage to primary care data for case ascertainment. We have not restricted diagnoses to a particular age and here, as in other cohorts ^5, 27^, linkage to primary care data has been essential for identifying large numbers of cases that were diagnosed after infancy. This is of particular importance for CHD diagnoses. Although CHD detection rates have improved in recent years in line with screening programs and technological advancements ^28^, there are still a proportion of cases that remain undiagnosed throughout early life and even into adulthood ^29^. These are likely to be less severe cases than those diagnosed antenatally or in infancy, but are important for unbiased studies of the causes, natural history and consequences of CHD. Linkage to primary care data in the UK (as in other countries) has been restricted until recently. It is appropriate that any such linkage is carefully controlled, for example through the use of a Trusted Research Environment for data storage and access, as we did through the use of ALSPAC’s UK Secure eResearch Platform (SeRP). However, our research shows the importance of being able to link to these data in just one field (CHDs). We have demonstrated the importance of linking original cohort data to external data sources such as primary health records to further strengthen the platform. A further advantage is that researchers can now link the CA data that we have identified and coded to information collected on the ALSPAC participants from preconception through to adulthood and beyond. This includes, but is not limited to parental characteristics, childhood health and wellbeing, social and educational background and future outcomes that may differ between those with and without CAs. These data will provide unique opportunities to a multitude of researchers involved with CA research. In addition to this, the second generation of the ALSPAC cohort (ALSPAC-G2) is now underway ^15^ providing scope for future linkage and unique research opportunities, including exploring secular and birth cohort trends in the incidence and prognosis of CAs, as well as intergenerational causes ^15^. CAs are prospectively collected in ALSPAC-G2 through extractions of data in antenatal, labour, neonatal and health visitor (children to age 5 years) records, parental questionnaires, linkage to ONS for deaths data and linkage to primary care data.

One limitation of this dataset is that we have not been able to successfully link to NHS Hospital Episode Statistics (HES) due to project restrictions that were in place by NHS digital at the time of data collation. An overhaul of the data sharing agreement was required, which is still ongoing at the time of writing. HES contains the records of all hospital admissions, outpatient appointments and Accident and Emergency depertment attendances at NHS hospitals in England ^30^. This database might have provided additional cases of CAs, though given the primary care linkage we may not have identified many additional cases via HES. There are currently (March 2020) 14,819 singletons and twins enrolled in ALSPAC, who were alive at 1 year and have not subsequently withdrawn from the study. We have linked 11,810 (80%) of these participants and so may have missed some cases. At least some of those who were not eligible to be linked because of dying should have been captured by other data sources. Participants who refuse data linkage could differ notably from those who do not, but the proportion of these (~3%) is too small to notably influence any analyses with these data. Failure to link to some of the eligible (for linkage) participants will mostly reflect those who are living outside the BNSSG area and/or registered with a practice that does not use the EMIS or Apollo clinical records system. As primary care data ‘follows the patient’, should any of these missing participants register with an eligible practice, then we may be able to link to additional records. Data on these participants (and any new CA diagnoses in later adulthood) would be obtained with future extractions. Furthermore, there are efforts to coordinate primary care record linkage for all cohorts across the UK. Thus, it may be possible for ALSPAC to extend linkages to additional participants as the infrastructure for primary care record linkage in the UK matures. We would update this data note with any future additional record linked data from primary care or HES.

Another limitation is that ALSPAC-G1 participants were born before the start of transition between paper and digital health records, and that fetal anomaly screening using ultrasound scans at 18–20 weeks was not yet advanced enough to capture most cases of CAs. Therefore, antenatal and early life health data that is available now was not available for this cohort. However, we have attempted to address this in our multi-source approach to defining cases, which includes data from antenatal, labour and postnatal data extractions by ALSPAC employed research midwives. Whilst contemporary cohorts, including ALSPAC-G2 are able to benefit from the availability of advances in the governance around linking cohorts to health records and the existence of extensive electronic health data, we believe the effort to collate and code the CA data in ALSPAC-G1 participants makes a key contribution to that study; given the extensive data available on these participants this provides a valuable research resource for ALSPAC-G2. Related to this, the enrolment period for ALSPAC-G1 participants (early 1990’s) predates the South West Congenital Anomaly Register (SWCAR) which began in 2002. The SWCAR was part of the British Isles Network of Congenital Anomaly registers and is now a member of Public Health England’s National Congenital Anomaly and Rare Diseases Registration Service (NCARDRS). Future data collections (e.g. in ALSPAC-G2 participants) should be cross-validated with these registers.

The descriptions above of each data source highlight their different coverage in terms of geography and time (participant age). They also vary between linkage to mortality and coded information in health records, detailed scrutiny and extraction of data from health records and a search of text entered by parents in questionnaires about their child. We have constructed the ALSPAC-G1 CA dataset by bringing all of these data together in an attempt to have not missed any cases whilst being as transparent as possible around the methods and data sources used. We feel that combining data in the way that we have provides the best estimate of CA in ALSPAC-G1. However, data are available with codes that clearly indicate their source, which enables any researcher who wanted to restrict main analyses to selected data sources only and/or undertake sensitivity analyses to explore whether results change if some datasets are not included. Researchers can also access and undertake analyses including (or comparing to) the 608 participants who we have defined as having ‘possible’ CA based on text in just one ALSPAC questionnaire.

To conclude, we have identified CAs in ALSPAC-G1 from multiple sources that are described here. The CAs have all been coded according to ICD-10 and are available to researchers (see *Data availability*). The linkage of these data to participants who are now in their late 20s and have a wealth of data from when they were in utero to the current time, including on their children as they start to become parents, makes this a powerful resource for CA research. The effort to obtain these should not be required for most contemporary birth cohorts given improved linkage systems and screening for CAs. However, it remains the case that CAs are under-researched and some birth cohorts exclude known CAs at recruitment. This may reflect concerns that within any single cohort cases may be too few for meaningful analyses. However, with birth cohorts increasingly collaborating and sharing data, for example as in the LifeCycle collaboration ^31^, the potential to generate sufficient numbers for analyses is possible and we would recommend cohorts do not exclude such patients and existing (older) cohorts like ALSPAC who have not previously tried to identify all cases do so.

## Data availability

### Underlying data

The ALSPAC data management plan describes in detail the policy regarding data sharing, which is through a system of managed open access. The steps below highlight how to apply for access to the data for all ALSPAC data and the data included in this paper.

1. Please read the ALSPAC access policy which describes the process of accessing the data and samples in detail, and outlines the costs associated with doing so: http://www.bristol.ac.uk/media-library/sites/alspac/documents/researchers/data-access/ALSPAC_Access_Policy.pdf.
2. You may also find it useful to browse the fully searchable ALSPAC research proposals database, which lists all research projects that have been approved since April 2011: https://proposals.epi.bristol.ac.uk.
3. Please submit your research proposal for consideration by the ALSPAC Executive Committee. You will receive a response within 10 working days to advise you whether your proposal has been approved.
4. Accessing the linked data used in this study will require ALSPAC to satisfy the governance requirements that accompany this use of health records (i.e. those imposed by the original data owners). All access to linked health records is via ALSPAC’s instance of the UK Secure Research Platform (UKSeRP): a remotely accessing secure research server. However, the intention is to make two variables available for use through the standard ALSPAC data application pathways. These two variables would include a) any congenital anomaly (yes/no) and b) any congenital heart disease (yes/no).

ALSPAC is a managed access resource, where the study charges users for the direct costs incurred when facilitating their research project. See here for more details: http://www.bristol.ac.uk/media-library/sites/alspac/documents/researchers/data-access/ALSPAC_Access_Policy.pdf.

If you have any questions about accessing data, please email alspac-data@bristol.ac.uk.

### Extended data

Open Science Framework: Ascertaining and classifying cases of congenital anomalies in the ALSPAC birth cohort. https://doi.org/10.17605/OSF.IO/NHZXY ^18^.

This project contains the following extended data:

- ALSPAC_CAs_ExtendedData.pdf: Table S1, The ICD and GP Read codes used to find possible case matches in the primary care data; Table S2, ALSPAC questionnaires and questions used for the child-based questionnaire category; Table S3, Search strategy for child-based questionnaires; Table S4, ICD-10 codes used to classify congenital anomalies; Table S5, ALSPAC cases included in 2 or more sources; Table S6, Remaining ALSPAC cases only included in 1 source.
- EUROCAT_Table_ALSPAC_DataNote.csv: EUROCAT prevalence’s used in Table 2.

Extended data are available under the terms of the Creative Commons Attribution 4.0 International license (CC-BY 4.0).
