# Supplementary material for: Ascertaining and classifying cases of congenital anomalies in the ALSPAC birth cohort
Source: Wellcome Open Res. 2021 Apr 14;5:231. Originally published 2020 Oct 6. [Version 2] doi: 10.12688/wellcomeopenres.16339.2 (PMC7871361; doi:10.12688/wellcomeopenres.16339.2)
Supplement: Peer review history

# Peer review: Reviewer response for version 1

**DOI:** 10.21956/wellcomeopenres.17965.r42019

**Published:** 2021-02-12

**Authors:** Judith Rankin

**Affiliations:** 1 Population Health Sciences Institute, Newcastle University, Newcastle-upon-Tyne, UK

This is a very well-written and detailed account of how individuals with a congenital anomaly were identified in the ALSPAC cohort. The ALSPAC is an amazing cohort and an important resource. The paper correctly refers to the need for more research on congenital anomalies as their aetiology is still largely unknown although some modifiable risk factors are well known. There is an extensive literature on different aspects of congenital anomalies but I accept that it’s not possible to include this in this type of methods paper.

- The data sources included congenital anomalies occurring in stillbirths and termination of pregnancy following prenatal diagnosis at any gestion which is best practice.
- I found figures 2 A & B very helpful in showing which data sources identified which cases.
- I think more detail is needed on how the congenital anomalies were categorised in table 2. So was each case counted once or each congenital anomaly in each case counted – this should be clarified in a footnote. It would also be helpful to know how many cases occurred in isolation and how many occurred in the presence of other anomalies. I am also not clear what the approach was to the inclusion or not of minor anomalies. EUROCAT recommends that minor anomalies occurring on their own should not be included ( Section 3.2- 27_Oct2016.pdf (europa.eu)).
- This paper relates to congenital anomalies in those individuals enrolled during 1990-92. This predates the South West Congenital Anomaly (SWCAR) register which began in 2002. The SWCAR was part of the British Isles Network of Congenital Anomaly registers and is now a member of Public Health England’s National Congenital Anomaly and Rare Diseases Registration Service (NCARDRS). This is a population-based, multisource register of congenital anomalies for England. The courses of data are too many to mention here but importantly, the governance is in place for NCARDRS to receive data feeds from the Office for National Statistics death registrations and Hospital Episode Statistics. Reference to NCARDRS should be made in the discussion and future cross-validation exercises undertaken.
- The focus on congenital heart diseases is understandable as they are the most common group of congenital anomalies, but it would also have been interesting to have included other groups to understand which data sources are the most efficient for which anomaly groups/subtypes.
- EUROCAT is the European Surveillance of Congenital Anomalies, this needs correcting.
- In the section on primary care data, it should be noted that not everyone is registered with a GP.
- It is important that the cohort is followed up so questions like the recurrence of congenital anomalies (s12916-017-0789-5.pdf ) can be investigated further.

Are sufficient details of methods and materials provided to allow replication by others?

Yes

Is the rationale for creating the dataset(s) clearly described?

Yes

Are the datasets clearly presented in a useable and accessible format?

Yes

Are the protocols appropriate and is the work technically sound?

Yes

Reviewer Expertise:

Congenital anomalies, registers, reproductive loss, maternal health, infant health

I confirm that I have read this submission and believe that I have an appropriate level of expertise to confirm that it is of an acceptable scientific standard.

# Response

**Published:** 2021-03-15

**Authors:** Kurt Taylor

**Affiliations:** University of Bristol, UK

**We thank the reviewer for their time to review this manuscript. The comments have been extremely useful in developing the manuscript. We include our responses below.**

  

- I think more detail is needed on how the congenital anomalies were categorised in table 2. So was each case counted once or each congenital anomaly in each case counted – this should be clarified in a footnote. It would also be helpful to know how many cases occurred in isolation and how many occurred in the presence of other anomalies. I am also not clear what the approach was to the inclusion or not of minor anomalies. EUROCAT recommends that minor anomalies occurring on their own should not be included (Section 3.2- 27_Oct2016.pdf (europa.eu)). 

**We thank the reviewer for bringing these important points to our attention. We have added the requested information to the footnote in Table 2. We have also added the following sentence under the subheading “Description of population”: “Of these 590 participants, 151 (25.6%) had a CA occurring in the presence of other anomalies.”**

  

- This paper relates to congenital anomalies in those individuals enrolled during 1990-92. This predates the South West Congenital Anomaly (SWCAR) register which began in 2002. The SWCAR was part of the British Isles Network of Congenital Anomaly registers and is now a member of Public Health England’s National Congenital Anomaly and Rare Diseases Registration Service (NCARDRS). This is a population-based, multisource register of congenital anomalies for England. The courses of data are too many to mention here but importantly, the governance is in place for NCARDRS to receive data feeds from the Office for National Statistics death registrations and Hospital Episode Statistics. Reference to NCARDRS should be made in the discussion and future cross-validation exercises undertaken. 

**We have added the following to the discussion section:**

**“Related to this, the enrolment period for ALSPAC-G1 participants (early 1990’s) predates the South West Congenital Anomaly Register (SWCAR) which began in 2002. The SWCAR was part of the British Isles Network of Congenital Anomaly registers and is now a member of Public Health England’s National Congenital Anomaly and Rare Diseases Registration Service (NCARDRS). Future data collections (e.g. in ALSPAC-G2 participants) should be cross-validated with these registers.“  **

- The focus on congenital heart diseases is understandable as they are the most common group of congenital anomalies, but it would also have been interesting to have included other groups to understand which data sources are the most efficient for which anomaly groups/subtypes. 

**We appreciate the reviewers understanding on our decision to focus on congenital heart diseases (CHDs) in this data note. As well as being the most common group of congenital anomalies, the focus on CHDs was also to align with the specific research interests of several of the authors including KT, MC and DAL. Indeed, these data in ALSPAC have recently contributed to important work exploring maternal risk factors for CHDs (paper under review: https://doi.org/10.1101/2020.09.29.20203786).**

  

- EUROCAT is the European Surveillance of Congenital Anomalies, this needs correcting. 

**Thank you. We have made this change. **

- In the section on primary care data, it should be noted that not everyone is registered with a GP. 

**We have added the following acknowledgement to the “NHS Primary Care Records” section:**

**“It is possible that some participants were or are not registered with a GP, although, we would expect this to be a small minority.”**

# Peer review: Reviewer response for version 1

**DOI:** 10.21956/wellcomeopenres.17965.r42070

**Published:** 2021-02-08

**Authors:** Lucia K. Dinarti

**Affiliations:** 1 Department of Cardiology and Vascular Medicine, Faculty of Medicine, Public Health and Nursing, Universitas Gadjah Mada, Dr. Sardjito Hospital, Yogyakarta, Indonesia

In this study, the authors tried to collect all major CA cases and found that CHD is the commonest CA that occurred in their population.

 This study is extremely complex and sophisticated. Well-established medical record either paper or electronic is the main

 requisite to perform this kind of study. However, in developing countries, as in Indonesia, where medical record storage is still a challenging issue, this study is almost impossible to conduct at this moment.

 However, we admit that this study will be a future model about the method to trace back all CA cases diagnosed in adulthood. Linkage medical records from National Insurance Database, Primary and Secondary Care Service and other health services are important to be developed in order to diagnose or to perform screening from early lifetime.

Are sufficient details of methods and materials provided to allow replication by others?

Yes

Is the rationale for creating the dataset(s) clearly described?

Yes

Are the datasets clearly presented in a useable and accessible format?

Yes

Are the protocols appropriate and is the work technically sound?

Yes

Reviewer Expertise:

Congenital Heart Defect

I confirm that I have read this submission and believe that I have an appropriate level of expertise to confirm that it is of an acceptable scientific standard.

# Peer review: Reviewer response for version 1

**DOI:** 10.21956/wellcomeopenres.17965.r42069

**Published:** 2021-01-26

**Authors:** Euiseok Jung

**Affiliations:** 1 Division of Neonatology, Department of Pediatrics, Asan Medical Center Children's Hospital, Seoul, South Korea

It's an honor to be given the opportunity to review a great cohort. This study is technically sound and well-written. I hope that the cohort will continue to progress through the 3rd and 4th generation in the future. As a result, I look forward to a review of studies with richer results. I would like to give you some opinions on the research. 

 1. Introduction:

- The content on congenital heart disease that was specifically highlighted in the abstract is missing. It would be better to give readers a good understanding of why authors decided to focus congenital heart disease. 

2. Description of population:

- According to the ALSPAC birth cohort, the prevalence of congenital heart disease is around 1%. This is similar to the prevalence of congenital heart disease in the United States. The study is likely to be enriched if a comparison of the prevalence of congenital heart disease by cohorts is also described.

Are sufficient details of methods and materials provided to allow replication by others?

Yes

Is the rationale for creating the dataset(s) clearly described?

Yes

Are the datasets clearly presented in a useable and accessible format?

Yes

Are the protocols appropriate and is the work technically sound?

Yes

Reviewer Expertise:

neonatal intensive care, congenital heart

I confirm that I have read this submission and believe that I have an appropriate level of expertise to confirm that it is of an acceptable scientific standard.

# Response

**Published:** 2021-03-15

**Authors:** Kurt Taylor

**Affiliations:** University of Bristol, UK

**We thank the reviewer for their time to review this manuscript. We include responses below.**

 1. Introduction: 

 The content on congenital heart disease that was specifically highlighted in the abstract is missing. It would be better to give readers a good understanding of why authors decided to focus congenital heart disease.  

**In the methods section under “Aims” we have included our rationale for focusing on CHDs:**

**“The focus on CHDs reflects the specific research interests of KT, MC and DAL and because CHDs are the commonest form of CAs.”**

 2. Description of population: 

 According to the ALSPAC birth cohort, the prevalence of congenital heart disease is around 1%. This is similar to the prevalence of congenital heart disease in the United States. The study is likely to be enriched if a comparison of the prevalence of congenital heart disease by cohorts is also described.  

**We have added a comparison with other European birth cohorts using some of our recent work as an example ( https://doi.org/10.1101/2020.09.29.20203786):**

**“The prevalence of CHDs in ALSPAC is similar to other European birth cohorts. In recent work involving 7 European birth cohorts, we have shown that the prevalence of CHD was close to 1% in most cohorts, with the lowest with 0.4% and the highest with 1.4%. Differences in case ascertainment could be one of a number of possible explanations for the slight differences in prevalence estimates.”**
